# Long-term Mortality Predictors in Patients with Small Aortic Annulus Undergoing Aortic Valve Replacement with a 19- or 21-mm Bioprosthesis

**DOI:** 10.5935/1678-9741.20160060

**Published:** 2016

**Authors:** Jenny Lourdes Rivas de Oliveira, Renato Tambellini Arnoni, Magaly Arrais dos Santos, Antonio Flávio Sanchez Almeida, Mário Issa, Antoninho Sanfins Arnoni, Paulo Chaccur, Luiz Carlos Bento de Souza

**Affiliations:** 1 Instituto Dante Pazzanese de Cardiologia, São Paulo, SP, Brazil.

**Keywords:** Aortic Valve Stenosis, Heart Valve Prosthesis Implantation, Bioprosthesis, Mortality

## Abstract

**Introduction:**

Replacement of the aortic valve in patients with a small aortic annulus is associated with increased morbidity and mortality. A prosthesis-patient mismatch is one of the main problems associated with failed valves in this patient population.

**Objective:**

To evaluate the long-term mortality predictors in patients with a small aortic annulus undergoing aortic valve replacement with a bioprosthesis.

**Methods:**

In this retrospective observational study, a total of 101 patients undergoing aortic valve replacement from January 2000 to December 2010 were studied. There were 81 (80.19%) women with a mean age of 52.81±18.4 years. Severe aortic stenosis was the main indication for surgery in 54 (53.4%) patients. Posterior annulus enlargement was performed in 16 (15.8%) patients. Overall, 54 (53.41%) patients underwent concomitant surgery: 28 (27.5%) underwent mitral valve replacement, and 13 (12.7%) underwent coronary artery bypass graft surgery.

**Results:**

Mean valve index was 0.82±0.08 cm^2^/m^2^. Overall, 17 (16.83%) patients had a valve index lower than 0.75 cm^2^/m^2^, without statistical significance for mortality (*P*=0.12). The overall 10-year survival rate was 83.17%. The rate for patients who underwent isolated aortic valve replacement was 91.3% and 73.1% (*P*=0.02) for patients who underwent concomitant surgery. In the univariate analysis, the main predictors of mortality were preoperative ejection fraction (*P*=0.02; HR 0.01) and EuroSCORE II results (*P*=0.00000042; HR 1.13). In the multivariate analysis, the main predictors of mortality were age (*P*=0.01, HR 1.04) and concomitant surgery (*P*=0.01, HR 5.04). Those relationships were statistically significant.

**Conclusion:**

A valve index of < 0.75 cm^2^/m^2^ did not affect 10-year survival. However, concomitant surgery and age significantly affected mortality.

**Table:** 

Abbreviations, acronyms & symbols	
CABG	= Coronary artery bypass graft
CI	= Confidence interval
CPB	= Cardiopulmonary bypass
EF	= Ejection fraction
LVEF	= Left ventricular ejection fraction
NYHA	= New York Heart Association
PPM	= Prosthesis-patient mismatch

## INTRODUCTION

Prosthetic aortic valve replacement is a therapeutic option for patients with symptomatic aortic valve disease. Patients with aortic stenosis benefit from aortic valve replacement because this procedure reduces left ventricular afterload, which leads to a significant reduction in left ventricular muscle hypertrophy and marked clinical improvement^[1]^.

In the late 1970s, Rahimtoola^[2]^ suggested that the main complications from valve replacement surgery were thromboembolism, bleeding from anticoagulation therapy, prosthetic dysfunction, the need for valve re-replacement, and prosthesis-patient mismatch (PPM). Additionally, complications arise when the effective orifice area of the implanted prosthesis is considered small in relation to the patient's body surface. This condition leads to severe hemodynamic disorders in patients, thereby triggering the exchange of one disease for another^[2]^.

Small aortic annulus is associated with increased operative mortality due to PPM, which results in significantly increased mortality in the short- and long-term^[3,4]^.

Aortic valve replacement candidates most likely to suffer from PPM are generally elderly patients with a small aortic root diameter or patients with left ventricular hypertrophy^[5]^. In addition, obesity is associated with increased late mortality and a poor quality of life in patients with a small aortic valve who undergo prosthetic valve replacement^[6]^.

Enlargement of the aortic annulus is a surgical option to reduce the risk of PPM and late mortality. However, several studies have shown that this procedure is associated with a significant increase in surgical risk^[7]^.

Calculating the valve index is recommended when selecting the size and type of prosthesis that will provide an adequate effective orifice for the patient's body surface. Studies have shown that for patients with a body surface area of less than 1.7 m^2^, it is safe to use a prosthesis smaller or equal to 21 mm^[2,8,9]^. However, studies suggest that the use of prostheses smaller than the recommended size does not result in higher residual gradients^[10]^.

Concomitant coronary artery bypass graft surgery (CABG) and advanced age are risk factors that affect the long-term survival of patients undergoing aortic valve replacement, and the risk increases when PPM occurs^[11]^.

The aim of the present study was to evaluate the long-term mortality predictors in patients with a small aortic annulus undergoing aortic valve replacement with a 19- or 21-mm bioprosthesis.

## METHODS

Between January 2000 and December 2010, a total of 1,559 prostheses were implanted in the aortic valve position. Of those, 165 were biological and either 19 or 21 mm in size. A total of 101 patients who underwent implantation of a 19- or 21-mm bioprosthesis with or without enlargement of the aortic annulus and who had complete medical records were included in this study.

Mean age of the patients was 52.81±18.4 years (12-81 years old, median 57 years old). A total of 81 (80.19%) patients were women, and most patients (81-80.1%) were classified as New York Heart Association (NYHA) functional class II or III. Surgery was indicated for significant stenosis in 54 (53.41%) patients, double aortic lesions in 27 (26.5%), and bioprosthetic dysfunction in 8 (7.8%). Overall, 3 patients were excluded because they were younger than 12 years of age. Mean follow-up time was 8.16 years (95% CI 7.40-8.93 years), with a maximum of 10 years.

The findings in the relevant preoperative echocardiograms were left ventricular ejection fraction (LVEF), mean 65.36±9.2% (27%-87%, median 66%); transvalvular aortic systolic gradient, mean 49.10±0.54 mmHg (4-104 mmHg, median 47 mmHg); left atrium >40 mm in 77 (76.23%) patients; moderate or severe pulmonary hypertension in 30 (29.70%) patients; and moderate or severe left ventricular hypertrophy in 34 (33.66%) patients. The mean EuroSCORE II finding was 5.15±4.35% (0.71%-35.50%, median 3.86%). EuroSCORE II was used to calculate risk instead of the EuroSCORE because its model is more updated, with better calibration and discrimination, especially for patients undergoing aortic valve replacement with concomitant procedures^[12]^. The demographics and clinical characteristics of the patients are reported in Table 1.

**Table 1:** Preoperative clinical characteristics of the patients.

Variables	Total population n=101	*P* value
Age, years	52.81±18.4	0.017
Body surface area, m^2^	1.59±0.15	0.20
EuroSCORE II	5.15±4.35%	0.00000042
SAH	57 (56.43%)	0.04
DM	19 (18.81%)	0.03
Dyslipidemia	31 (30.69%)	0.15
Peripheral arterial disease	6 (5.94%)	0.03
Kidney failure	5 (4.95%)	0.03
COPD	8 (7.92%)	0.69
Current smoker	17 (16.83%)	0.49
Rheumatic fever	30 (29.70%)	0.19
Prior heart surgery	30 (29.70%)	0.68
Obesity	9 (8.91%)	0.12
NYHA II-III	81 (80.19%)	0.26
Ejection fraction < 60%	14 (13.86%)	0.02

SAH=systemic arterial hypertension; DM=diabetes mellitus; COPD=chronic obstructive pulmonary disease; NYHA=New York Heart Association

Implantation of the 19- and 21-mm biological valve prostheses in the aortic position was performed using median sternotomy with extracorporeal circulation and moderate hypothermia of 30°C-32°C. Myocardial protection was achieved with crystalloid cardioplegia or hypothermic antegrade blood in the aortic root or directly in the coronary ostia depending on the competence of the aortic valve. In 16 (15.84%) patients, posterior enlargement of the aortic annulus was performed (Manouguian technique)^[13]^. The following types of bioprostheses were used: Biocor bovine pericardium in 78 patients, Biocor Epic in 15, Labcor in 4, and Braile in 2. A 21-mm prosthesis was used in 99 patients and a 19-mm prosthesis was used in 2 (Biocor bovine pericardium).

The valve index was calculated for all patients by dividing the internal area of the prosthesis by the body surface. An index below 0.75 cm^2^/m^2^ was indicative of a risk of PPM^[2]^.

The data were collected retrospectively from clinical, surgical, and preoperative complementary tests and postoperative records. The analysis included 30-day and long-term postoperative evaluations of mortality and adverse events related to the surgical procedures.

The valve index in the deceased and non-deceased patients was evaluated. In addition, the final mean transvalvular aortic gradient relative to the valve index and survival for isolated aortic valve replacement and concomitant procedures were assessed.

The data are presented as frequency distribution and simple percentages. Continuous variables are expressed as mean ± standard deviation, and median when indicated. Categorical variables are expressed as absolute and relative frequencies. For the survival analysis and mortality predictors, we used the Kaplan-Meier curve method (with the log-rank test), Cox regression model, and the Mann-Whitney test. The variables that were significant in the univariate analysis or associated with clinical relevance were subsequently adjusted in the Cox multivariate analysis. The 95% confidence intervals (CIs) were calculated. *P*<0.05 was considered statistically significant.

## RESULTS

Aortic valve replacement with a 19- or 21-mm bioprosthesis was performed without annulus enlargement in 85 (84.15%) patients and with aortic annulus enlargement using the Manouguian technique in 16 (15.84%) patients. Of those patients, 54 (53.46%) underwent concomitant surgery, including mitral valve replacement in 28 (27.50%) patients and myocardial revascularization in 13 (12.70%). Mean anoxia time was 80.52±7.15 minutes (36-160 minutes, median 76 minutes), and mean cardiopulmonary bypass (CPB) time was 114.44±48.19 minutes (45-325 minutes, median 100 minutes).

Mean valve index was 0.82±0.08 cm^2^/m^2^ (0.65-1.18 cm^2^/m^2^, median 0.83 cm^2^/m^2^), and 17 (16.83%) patients had a valve index lower than 0.75 cm^2^/m2 (*P*=0.12).

**Fig. 1:**
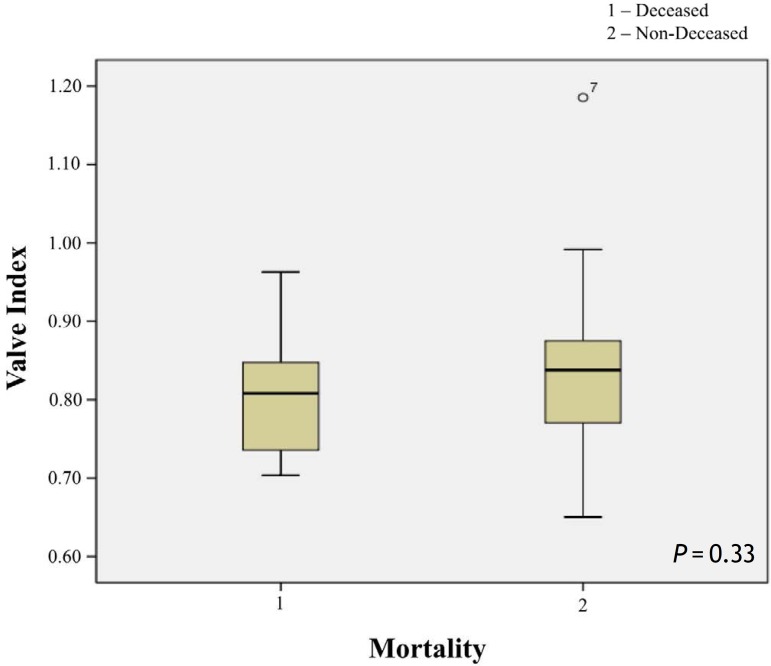
Valve index and mortality.

No differences were found between the valve indices in the deceased and non-deceased patients. The mean valve index for the deceased patients was 0.81±0.006 cm^2^/m^2^ (0.76-0.85 cm^2^/m^2^, median 0.80 cm^2^/m^2^) and the mean index for the non-deceased patients was 0.82 cm^2^/m^2^±0.007 (0.81-0.84 cm^2^/m^2^, median 0.82 cm^2^/m^2^) (P=0.33) (Figure 1).

Mean preoperative aortic transvalvular gradient was 49.10±20.54 mmHg (4-104 mmHg, median 47.00 mmHg). In the early postoperative period, the mean gradient was 20.54±7.55 mmHg (5-42 mmHg, median 20.00 mmHg), and the long-term mean gradient was 26.11±12.72 mmHg (5-64 mmHg, median 23 mmHg). Overall, 1 (0.99%) patient had a mean aortic transvalvular gradient greater than 40 mmHg after aortic valve replacement and a valve index of 0.72 cm^2^/m^2^, which suggests a PPM. A lower valve index was associated with a higher mean postoperative aortic transvalvular gradient. However, this finding was not significant (*P*=0.20).

**Fig. 2:**
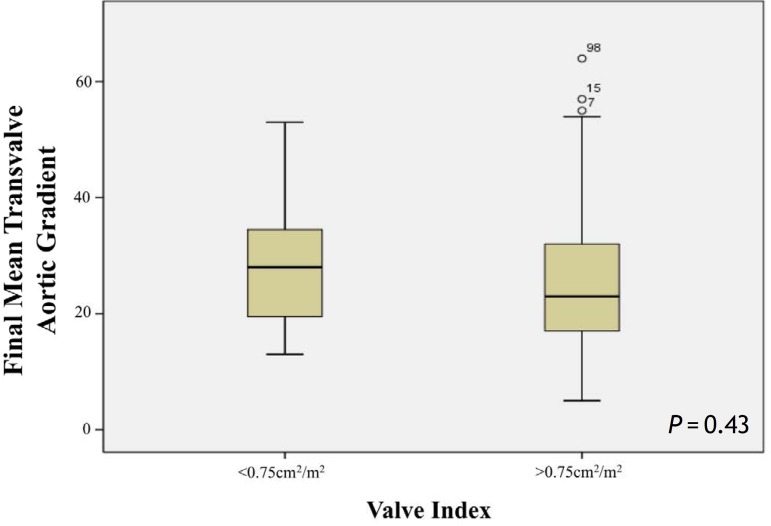
Valve index and final mean systolic gradient.

In the long-term analysis, when the valve index was < 0.75 cm^2^, the final mean aortic transvalvular valve gradient was 28.64±12.40 mmHg (13-53 mmHg, median 28 mmHg). When the valve index was >0.75 cm^2^, the mean gradient was 25.71±12.80 mmHg (5-64 mmHg, median 23 mmHg); however, this finding was not significant (*P*=0.43) (Figure 2). The 30-day postoperative results are reported in Table 2.

**Table 2:** Postoperative results at 30 days.

Variables	Total population n=101
Inotropic support	14 (13.86%)
IABP	5 (4.95%)
Reoperation for bleeding	5 (4.95%)
Respiratory complications	10 (9.90%)
Stroke	4 (3.96%)
Permanent pacemaker	1 (0.99%)
Hospitalization time	9.0±5.0 days
30-day mortality	15 (14.85%)

IABP=intra-aortic balloon pump

A total of 27 patients had complications during hospitalization; 8 (7.92%) patients had pneumonia, and 4 (3.96%) had atrial fibrillation with a rapid ventricular response.

**Fig. 3:**
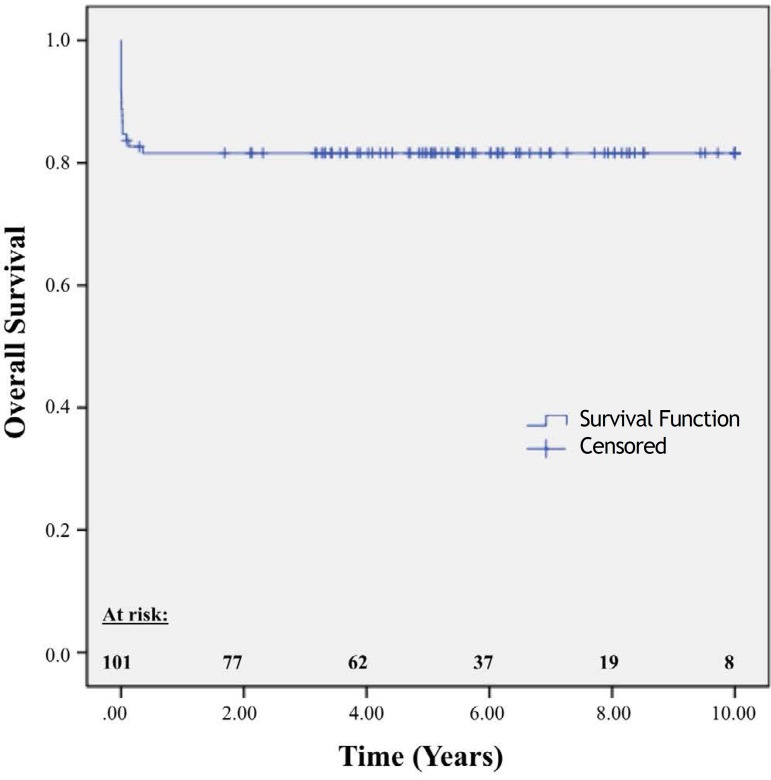
Kaplan-Meier survival curve of patients undergoing aortic valve replacement with a bioprosthesis (19 and 21 mm).

**Fig. 4:**
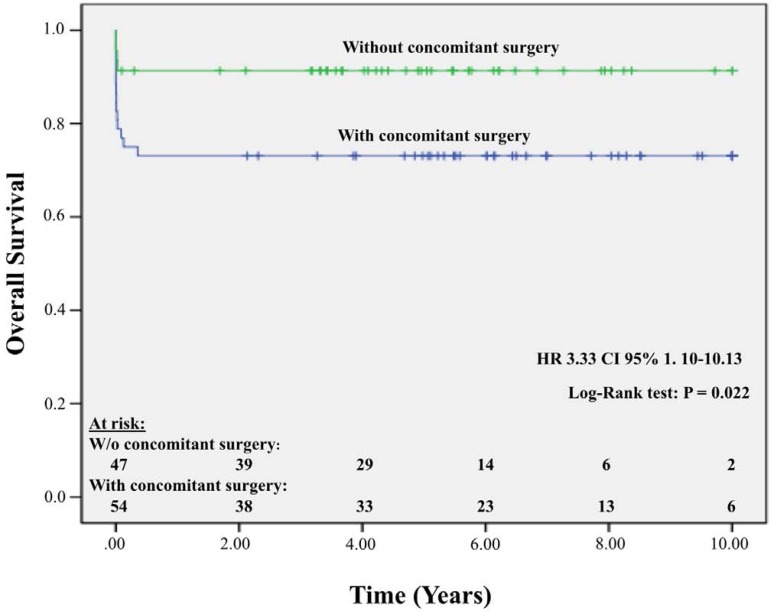
Survival curve of patients undergoing aortic valve replacement with and without concomitant surgery.

The overall 10-year survival rate was 83.17% (Figure 3). The 10-year survival rate for patients who underwent isolated aortic valve replacement was 91.3%, and the rate for patients who underwent concomitant surgery was 73.1% (*P*=0.02). The difference in survival rates was detected during the first 6 months of follow-up because 15 of the 17 patient deaths occurred in the first 30 days (Figure 4).

In the univariate analysis, the main mortality predictors were preoperative ejection fraction (EF) (*P*=0.02; HR 0.01, 95% CI 0.0002-0.53) and EuroSCORE II results (*P*=0.00000042; HR 1.13, 95% CI 1.08-1.19).

NYHA functional class (*P*=0.19; HR 1.87, 95% CI 0.72-4.82) and reoperation (*P*=0.67, HR 1.23, 95% CI 0.46-3.28) were not statistically significant.

In the multivariate analysis, when age, valve index, concomitant surgery, and EF were analyzed simultaneously, only age (*P*=0.01, HR 1.04, 95% CI 1.009-1.08) and concomitant surgery (*P*=0.01; HR 5.04, 95% CI 1.41-18.02) were significant predictors of mortality (Table 3).

**Table 3:** Multivariate analysis.

Variables	*P* value	HR	CI 95.0% for HR	
			Minimum	Maximum
Age	0.014	1.046	1.009	1.083
Valve index	0.152	0.459	0.158	1.331
Additional surgery	0.013	5.046	1.412	18.026
EF	0.079	0.016	0.000	1.608

EF=ejection fraction; HR=Hazard Ratio

**Fig. 5:**
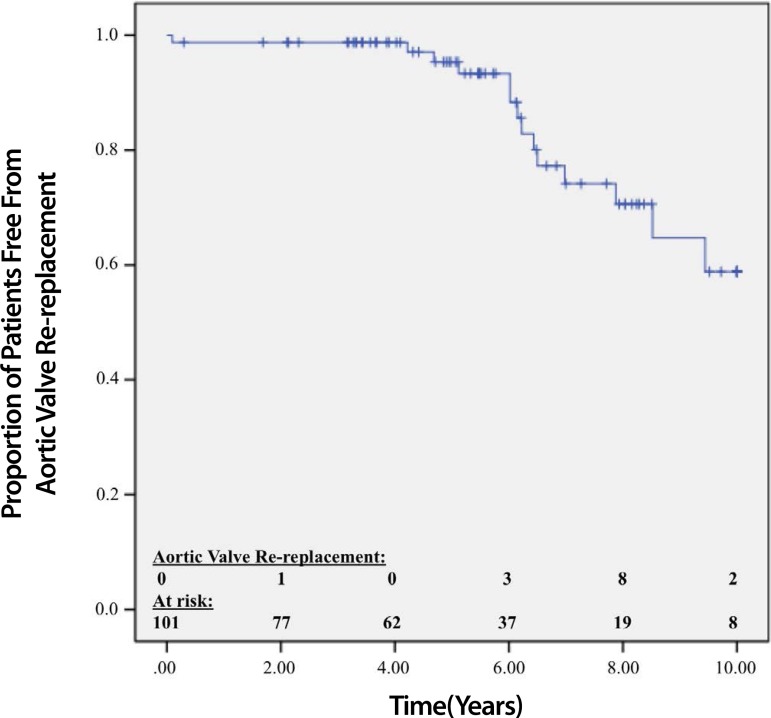
Kaplan-Meier curve shows the time until aortic valve rereplacement was needed.

During 10 years of follow-up, 14 patients required aortic valve re-replacement. Of those, re-replacement was indicated for bioprosthesis dysfunction in 10 (71.42%) patients, endocarditis in 3 (21.42%) patients, and PPM in 1 (7.14%). After 4 years of follow-up, 98% of the patients did not require aortic valve rereplacement. After 5 years of follow-up, 95.3% of the patients did not require aortic valve re-replacement, and after 10 years, 58.8% of the patients did not require valve re-replacement (Figure 5).

## DISCUSSION

A small aortic annulus is predominantly found in female and elderly patients; therefore, comorbidities and increased risks are associated with these patient populations^[4,8,14-16]^.

Bahlmann et al.^[17]^ evaluated a total of 1,563 patients with mild to moderate aortic stenosis and found a small aortic annulus in 32% of the patients, which was twice as frequently found in women (*P*<0.05). A small aortic annulus is an independent predictor of mortality in patients with aortic stenosis^[17]^.

In our study, 80.19% of the patients were women. However, the mean age of our patients was 52.81±18.4 years, which is lower than the mean age found in other studies^[14,15]^.

Previous studies have shown a low frequency of patients with severe PPM and a valve index lower than 0.65 cm^2^/m^2^^[10,16,18,19]^. In the present study, only 17 (16.83%) patients had a valve index lower than 0.75 cm^2^/m^2^, and only 1 (0.99%) patient had severe PPM requiring aortic valve re-replacement.

In studies that evaluated the impact of concomitant surgeries on outcomes of aortic valve replacement, CABG was the most common concomitant procedure^[11,18,20,21]^. In our study, the most common concomitant procedures were mitral valve replacement (27.5% of patients) and myocardial revascularization (12.7% of patients).

A study of 148 patients who underwent isolated aortic valve replacement with a 19- or 21-mm bioprosthesis revealed a 30- day mortality rate of 6.1%^[14]^. Another study of 68 patients who received an 18- or 20-mm Sorin Soprano prosthetic implant showed a 30-day mortality of 4.4%^[22]^. In a series of 53 patients undergoing aortic valve replacement plus annulus expansion with implantation of a 19-, 21-, or 23-mm prosthesis, the 30-day mortality rate was 2%^[23]^. Another study found that the 30-day mortality rate was 4.3% in patients who received implanted prostheses smaller than 22 mm^[7]^.

In a study of 41 patients who received either a stentless aortic prosthesis or a conventional prosthesis with or without CABG, Rao et al.^[20]^ obtained a mortality rate of 0% in patients with a stentless prosthesis *versus* 6% in patients with a conventional prosthesis.

Another study that evaluated mechanical prosthetic valves implanted in the aortic position found an in-hospital mortality rate of 3.9%^[24]^. In a study of 11 female patients who received 17-mm Regent St. Jude metallic prostheses, Takaseya et al.^[19]^ observed a 0% mortality rate.

Our 30-day mortality rate for isolated aortic valve replacement (1.98%) is lower than those in other studies. This finding is most likely because the mean age of our population was lower and their etiology was usually rheumatic. However, the mortality rate increased to 12.87% (*P* =0.02) in patients who underwent concomitant surgery because this patient group is at a higher risk of mortality due to longer operative and CPB times.

In a retrospective study of elderly patients undergoing aortic valve replacement, Tagliari et al.^[25]^ found an in-hospital mortality rate of 9.4% in the isolated aortic stenosis surgery group *versus* 20.9% in patients who underwent another surgical procedure. The main mortality predictors were ischemia time > 90 minutes, EF < 60%, and prior stroke.

Regarding long-term survival rates following aortic valve replacement, a study of high-risk patients^[14]^ found a 10-year survival rate of 40.9%. Celiento et al.^[23]^ found a 10-year survival rate of 68±7% in patients who underwent valve replacement with aortic annulus enlargement.

In our study, the 10-year survival rate was 83.17%. This finding was comparable with the results obtained by Walther et al.^[18]^ who observed a survival rate of 79.6±1.3% in patients with PPM *versus* 84.9±0.7% in patients without PPM (P<0.01). Additionally, our study showed no statistically significant relationship between PPM and mortality (*P*=0.12).

Several studies have shown a significant association between PPM and mortality^[3,4,16]^. A study demonstrated a correlation between the valve index and mortality: a higher mortality rate was associated with a lower valve index. In addition, the 8-year survival rates were 41%, 65%, and 74% when the valve index was <0.60 cm^2^/m^2^, between 0.60 and 0.85 cm^2^/m^2^, and <0.85 cm^2^/m^2^, respectively^[4]^.

Similar to our study, Howell et al.^[21]^ showed no significant association between PPM and mortality in two different studies. In the prospective study of 1,481 patients with or without CABG, the 5-year survival was similar in the PPM and non-PPM groups (83% *vs.* 81%, respectively, *P*=0.47). In another study of 801 patients who underwent isolated aortic valve replacement, PPM was not an independent mortality risk factor in either the short- or long-term for moderate (*P*=0.4; HR 1.12) or severe (*P*=0.92; HR 0.94) PPM^[16]^.

In a multivariate analysis of mortality predictors in patients undergoing aortic valve replacement surgery, He et al.^[11]^ concluded that older age (*P*=0.0061; HR 1.0258) and concomitant CABG (*P*=0.0115; HR 1.7146) were independent risk factors that affected long-term survival. They observed a 10-year survival rate of 71% for valve replacement without CABG and 40% when valve replacement was associated with this procedure (*P*=0.02). In a Cox regression, Howell et al.^[21]^ identified age as the only significant predictor of mortality (*P*=0.004; RR 2.13). In another study by Howell et al.^[16]^, only the EuroSCORE findings were a significant short- and long-term independent risk factor.

Those previous data are comparable to our study, in which the univariate analysis showed that preoperative EF (*P*=0.02; HR 0.01) and EuroSCORE II results (P=0.00000042; HR 1.13) were significant predictors of mortality, and the multivariate analysis showed that age (*P*=0.01, HR 1.04) and concomitant surgery (*P*=0.01, HR 5.04) were significant independent predictors of mortality.

Because of limited control in obtaining the patient sample, only patients who underwent outpatient monitoring at the study institution were included. Therefore, this study represents the experience of a single institution and cannot be generalized to all patients with a small aortic annulus. In addition, the surgical procedures were performed by different surgeons with various levels of experience.

## CONCLUSION

Implantation of a 19- or 21-mm bioprosthesis in patients undergoing aortic valve replacement is a safe procedure. A valve index < 0.75 cm^2^/m^2^ was found in 16.83% of the patients, but it was not significantly associated with increased mean aortic valve gradient in either the short or long-term nor was it associated with a higher mortality in either the short or long term. Concomitant surgery, age, EF, and EuroSCORE II findings were the main mortality predictors and significantly affected short-term survival. After 6 months of follow-up, no differences were observed in the survival rates.

**Table:** 

Authors’ roles & responsibilities	
JLRO	Conception and study design; execution of operations and/or trials; analysis and/or data interpretation; statistical analysis; manuscript writing or critical review of its content; final manuscript approval
RA	Execution of operations and/or trials; analysis and/or data interpretation; manuscript writing or critical review of its content; final manuscript approval
MAS	Execution of operations and/or trials; analysis and/or data interpretation; manuscript writing or critical review of its content; final manuscript approval;
AFSA	Execution of operations and/or trials; final manuscript approval
MI	Execution of operations and/or trials; final manuscript approval
AA	Execution of operations and/or trials; final manuscript approval
PC	Execution of operations and/or trials; final manuscript approval
LCBS	Execution of operations and/or trials; final manuscript approval
